# Potential Anti-HIV Agents from Marine Resources: An Overview

**DOI:** 10.3390/md8122871

**Published:** 2010-11-29

**Authors:** Thanh-Sang Vo, Se-Kwon Kim

**Affiliations:** 1 Marine Biochemistry Laboratory, Department of Chemistry, Pukyong National University, Busan 608-737, Korea; E-Mail: sanghooklee@yahoo.com; 2 Marine Bioprocess Research Center, Pukyong National University, Busan 608-737, Korea

**Keywords:** anti-HIV agents, AIDS, phlorotannins, marine resources

## Abstract

Human immunodeficiency virus (HIV) infection causes acquired immune deficiency syndrome (AIDS) and is a global public health issue. Anti-HIV therapy involving chemical drugs has improved the life quality of HIV/AIDS patients. However, emergence of HIV drug resistance, side effects and the necessity for long-term anti-HIV treatment are the main reasons for failure of anti-HIV therapy. Therefore, it is essential to isolate novel anti-HIV therapeutics from natural resources. Recently, a great deal of interest has been expressed regarding marine-derived anti-HIV agents such as phlorotannins, sulfated chitooligosaccharides, sulfated polysaccharides, lectins and bioactive peptides. This contribution presents an overview of anti-HIV therapeutics derived from marine resources and their potential application in HIV therapy.

## 1. Introduction

Human immunodeficiency virus type-1 (HIV-1) is the cause of acquired immune deficiency syndrome (AIDS), a major human viral disease with about 33.2 million people infected worldwide up to now [1,2]. Antiviral agents that interfere with HIV at different stages of viral replication have been developed [3,4]. However, failure in anti-AIDS treatment is observed due to the emergence of resistant viruses, cross-resistance to drugs and cell toxicity [5,6]. Therefore, the search for potential drug candidates containing higher inhibitory activity against various HIV strains is increasing in the pharmaceutical industry. In this regard, natural bioactive compounds and their derivatives are great sources for the development of new generation anti-HIV therapeutics which are more effective with fewer side-effects [7–9].

In the past, the screening and development of natural products and chemically synthesized compounds have been developed as medication for HIV infections [10–14]. With marine species comprising approximately one-half of the total global biodiversity, the sea offers an enormous resource for novel compounds [15]. Moreover, very different kinds of substances have been procured from marine organisms because they are living in a very exigent, competitive and aggressive surrounding; very different in many aspects from the terrestrial environment, a situation that demands the production of quite specific and potent active molecules. The marine environment serves as a source of functional materials, including polyunsaturated fatty acids (PUFA), polysaccharides, minerals and vitamins, anti-oxidants, enzymes and bioactive peptides [16,17]. This paper focuses on anti-HIV therapeutic agents derived from marine resources and their potential medicine/medical application as novel functional ingredients in anti-HIV therapy.

## 2. Potential Marine-Derived Anti-HIV Agents and Their Anti-HIV Activity

### 2.1. Phlorotannins

**Figure 1:**
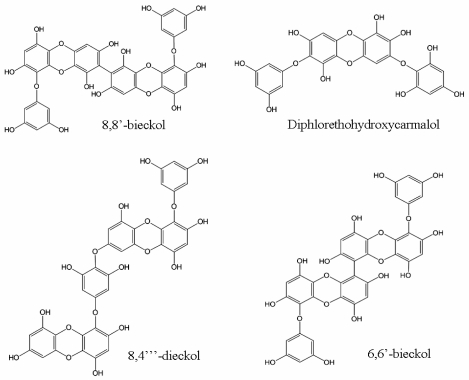
Chemical structure of phlorotannins.

Phlorotannins, which have been found to exist within brown algae, are formed by the polymerization of phloroglucinol (1,3,5-tryhydroxybenzene) monomer units and biosynthesized through the acetatemalonate pathway [18,19]. The phlorotannins are highly hydrophilic components with a wide range of molecular sizes ranging between 126 Da–650 kDa [20]. Marine brown algae accumulate a variety of phloroglucinol-based polyphenols, as phlorotannins of low, intermediate and high molecular weight containing both phenyl and phenoxy units [21,22]. Furthermore, phlorotannins consist of phloroglucinol units linked to each other in various ways, and are of wide occurrence amongst marine organisms, especially brown and red algae [21]. Based on the means of linkage, phlorotannins can be classified into four subclasses: fuhalols and phlorethols (phlorotannins with an ether linkage), fucols (with a phenyl linkage), fucophloroethols (with an ether and phenyl linkage), and eckols (with a dibenzodioxin linkage) [23]. Brown algae, which have a high concentration of phlorotannins (Figure 1), have been reported to possess anti-HIV activity (Table 1).

For the first time, Ahn *et al*. [24] reported that 8,8′-bieckol and 8,4‴-dieckol, which were isolated from the brown algae *Ecklonia cava* KJELLMAN, show an inhibitory effect on HIV-1 reverse transcriptase and protease. The inhibition against reverse transcriptase of 8,8′-bieckol with a biaryl linkage (IC_50_, 0.5 μM) was 10-fold higher than that of 8,4‴-dieckol with a diphenyl ether linkage (IC_50_, 5.3 μM), although these two phlorotannis are dimers of eckol. The authors suggested that the steric hindrance of the hydroxyl and aryl groups near the biaryl linkage of 8,8′-bieckol caused the potent inhibitory activity. Moreover, 8,8′-bieckol selectively inhibited reverse transcriptase over protease, and the inhibitory effect was comparable to the positive control nevirapine (IC_50_, 0.28 μM). It is clear that the 8,8′-bieckol possessed higher inhibitory activity than 8,4‴-dieckol. Therefore, they evaluated the molecular mechanisms of this compound against HIV-1 reverse transcriptase using a homopolymeric template/primer under steady-state condition. Kinetic study showed that 8,8′-bieckol inhibited the RNA-dependent DNA synthesis activity of HIV-1 reverse transcriptase noncompetitively against dUTP/dTTP with a *K*i value of 0.78 μM. Meanwhile, this compound also exhibited a noncompetitive inhibition (*K*i, 0.23 μM) with respect to a homopolymeric template/primer, (rA)_n_(dT)_15_. A possible suggestion for this phenomenon is that 8,8′-bieckol binds to HIV-1 reverse transcriptase only after the template/primer initially binds to the enzyme. The inhibitory effects of this compound shown in this kinetic model are consistent with non-nucleoside RT inhibitors, such as pyridinones [27], trovirdine [28]. As a result, 8,8′-bieckol might be considered as a new nonnucleoside HIV-1 RT inhibitor.

In the next report, Ahn *et al*. [25] showed that *Ishige okamurae* Yendo-derived diphlorethohydroxycarmalol also has an inhibitory effect on HIV-1. This compound exhibited inhibitory effects on the HIV-1 reverse transcriptase and integrase with IC_50_ values of 9.1 μM and 25.2 μM, respectively. However, diphlorethohydroxycarmalol did not show inhibitory activity against the HIV-1 protease.

Furthermore, 6,6′-bieckol, one of the main phloroglucinol derivative naturally occurring in *Ecklonia cava*, has a potent inhibition against HIV-1 induced syncytia formation, lytic effects, and viral p24 antigen production [26]. Moreover, 6,6′-bieckol selectively inhibited the activity of HIV-1 reverse transcriptase enzyme with an IC_50_ of 1.07 μM, as well as the inhibition of HIV-1 entry. In addition, it exhibited no cytotoxicity at a concentration where it inhibited HIV-1 replication almost completely. Therefore, 6,6′-bieckol can be employed for the development of new generation therapeutic agents against HIV.

### 2.2. Chitin, Chitosan and Chitooligosaccharide Derivatives

**Figure 2:**
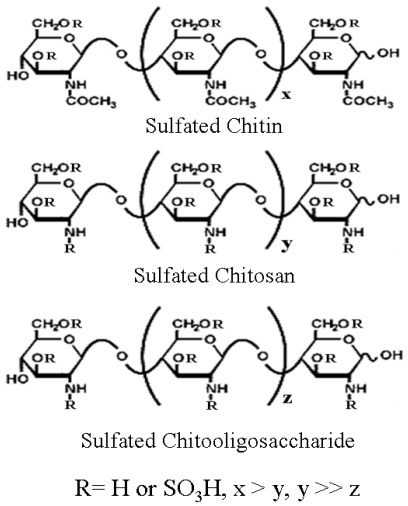
Chemical structure of sulfated chitin, chitosan and chitooligosaccharide.

Chitin, a long-chain polymer of *N*-acetylglucosamine, is widely distributed as the principle component of living organisms such as insects, fungi, crustacean and invertebrates [29]. It is one of the most abundant polysaccharides and is usually prepared from the shells of crabs and shrimps [30]. Chitosan, a partially deacetylated polymer of *N*-acetylglucosamine, is produced commercially by deacetylation of chitin [31]. Chemical modification of chitin and chitosan generate new biofunctional materials which provide desired biological activities and physicochemical properties [32–36]. The sulfated chitin and chitosan (Figure 2) have a variety of biological functions, including anti-HIV-1, anti-oxidant, anti-microbial, blood anti-coagulant and hemagglutination inhibition activities. Moreover, some of these sulfated chitin and chitosan derivatives function in drug delivery, adsorption of metal ions, prevention of cancer metastasis, or as elicitors of resistance to late blight in potato [37–40].

Sosa *et al*. [41] report that the *N*-carboxymethylchitosan *N*,*O*-sulfate (NCMCS), a polysaccharide derived from *N*-carboxymethyl chitosan by a random sulfation reaction, could inhibit the propagation of the human immunodeficiency virus type 1 (HIV-1) in human CD4^+^ cells. Furthermore, they suggested that this activity was due to blocking of the interactions of viral coat glycoprotein receptors to target proteins on lymphocytes and competitive inhibition of HIV-1 reverse transcriptase. Therefore, NCMCS is considered as a potent anti-infection agent, which prevents the invasion of HIV-1 through inhibiting viral adsorption to the CD4 receptor and reverse transcription of the viral genome. However, the inhibitory effect of chitin sulfates on HIV-1 infection depends significantly on the sites of sulfation (Table 2) [42]. A regioselective sulfation of C-2 (C-2S) and/or C-3 (C-3S) groups of chitin showed a much higher inhibitory effect on the infection of AIDS virus *in vitro* than the 6-*O*-sulfonated (C-6S) derivatives. Moreover, the product with sulfation at both 2 and 3 positions (C-2,3S) completely inhibited the infection of AIDS virus to T lymphocytes at a concentration of 0.28 μg/mL without significant cytotoxicity. These results indicate that biological activities of sulfated chitin are controllable by changing the position of the sulfate groups.

Recently, many studies have developed aminoderivatized chitosans, which possess numerous biological activities such as anti-oxidant, anti-hypertensive, enzyme inhibition, and anti-microbial properties [43]. Among aminoderivatized chitosans, aminoethyl-chitosan, prepared from 50% deacetylated chitosan, showed activity against HIV-1 with an IC_50_ value of 17 μg/mL [44]. Thus, aminoethyl-chitosan can be used as new generation drug candidates against HIV.

To improve the water-solubility and biological activity, chitosan also can be converted to chitooligosaccharides (COSs) via either chemical or enzymatic hydrolysis [45–47]. COSs and their derivatives are not only water-soluble [48] and have higher absorption profiles [49], but also possess various biological activities, such as ACE enzyme inhibition [50], anti-oxidant [51], anti-microbial [52], anti-cancer [53,54], immuno-stimulant [55], anti-diabetic [56], hypocholesterolemic [57], hypoglycemic [58], anti-Alzheimer’s [59], anti-coagulant [60] and adipogenesis inhibition [61]. In addition, sulfated chitooligosaccharide (SCOS), which were synthesized by a random sulfation reaction, have reported to possess anti-HIV activity at low molecular weight (3–5 kDa) (Figure 2) [62]. At nontoxic concentrations, SCOS significantly inhibited HIV-1-induced syncytia formation and lytic effect at EC_50_ values of 2.19 μg/mL and 1.43 μg/mL, respectively. As well, the production of p24 antigen was suppressed at EC_50_ values of 4.33 μg/mL and 7.76 μg/mL for HIV-1_RF_ and HIV-1_Ba-L_, respectively. Moreover, SCOS exhibited inhibitory activities on viral entry and virus-cell fusion via blocking the binding between HIV-1 gp120 and CD4 cell surface receptor. These observations indicate that SCOS might be useful as a novel candidate for the development of anti-HIV-1 agents.

### 2.3. Sulfated Polysaccharides

Sulfated polysaccharides (SPs) comprise a complex group of macro-molecules with a wide range of important biological activities. These polymers are chemically anionic and distributed widely not only in marine algae but also in animals such as mammals and invertebrates [63,64]. Marine algae are the most important source of non-animal SPs and the chemical structure of the polymers varies according to the algae species [65]. The amount of SPs in algae varies according to the divisions of marine algae, such as Chlorophyta (green algae), Rhodophyta (red algae) and Phaeophyta (brown algae). In recent years, various SPs isolated from marine algae have attracted much attention in the fields of biochemistry and pharmacology. They exhibit beneficial biological activities such as anti-HIV-1 [9], anti-adhesive [66], anti-coagulant [67], anti-cancer [68] and anti-inflammatory [69].

Many species of marine algae contain significant quantities of complex structural SPs that have been shown to inhibit the replication of enveloped viruses including members of the flavivirus, togavirus, arenavirus, rhabdovirus, orthopoxvirus, and herpesvirus families [70]. The chemical structure, including the degree of sulfation, molecular weight, constituent sugars, conformation and dynamic stereochemistry, affect the antiviral activity of algal sulfated polysaccharides [71–73]. Moreover, SPs may inhibit the attachment of viruses with target molecules on the cell surface. The viral attachment peptides are highly conserved regions within rather variable scaffolds of viral surface glycoproteins. These peptides are poorly subject to alterations by the natural antigenic drift of viruses. Likewise, they are not expected to represent frequent sites of drug-induced resistant mutation. Therefore, SPs directed toward these target peptides are preferred candidates for antiviral drug development [71,74–76]. In this regard, marine-derived SPs are great sources for the development of a new generation of anti-HIV therapeutics, as reported by several studies (Table 3). A number of SPs from red algae have exhibited an appreciable HIV-1 inhibitory activity. The sulfated glucuronogalactan from red algae *Schizymenia dubyi* was reported to possess anti-HIV activity [77]. Bourgougnonl *et al.* [77] determined the antiviral activity with HIV-1 by measuring the protective effect of sulfated glucuronogalactan against the virus-induced cytopathogenicity in MT4 cells over eight days. As shown in their study, the syncitial formation was completely suppressed with 5 μg/mL of this polysaccharide. Furthermore, the HIV-1 reverse transcriptase was inhibited at concentrations as low as 5 μg/mL, without cytotoxicity to MT4 cells. They suggested that the mechanism of action of this polysaccharide *in vitro* can be been mainly attributed to the inhibition of virus-host cell attachment or an early step of HIV infection.

In addition, sulfated galactans GFP extracted from the red algae *Grateloupia filicina* and GLPE obtained from *Grateloupia longifolia* also have antiretroviral activity *in vitro*. The sulfated galactan GFP has sulfate ester groups at carbon 2 and at carbon 2 and 6 for GLPE. Wang *et al*. [83] investigated the antiretroviral activity of these sulfated galactans in a model based on a primary isolate of HIV-1 and human peripheral blood mononuclear cells. These results showed that both GFP and GLPE had potent anti-HIV-1 activity when added at the time of infection and 2 h post-infection (EC_50s_, 0.010–0.003 μM and EC_90s_, 0.87–0.33 μM, respectively) with low cytotoxicity.

Moreover, brown algae are also known to produce a variety of interesting SPs, which have been found to exhibit anti-HIV activity with different mechanisms of action. Sulfated polymannuroguluronate (SPMG), a new form of sulfated polysaccharide extracted from brown algae with an average molecular mass of 8.0 kDa, is rich in 1,4-linked b-D-mannuronate, with an average of 1.5 sulfates and 1.0 carboxyl groups per sugar residue [86]. The involution of marine sulfated polymannuroguluronate in inhibition of HIV-1 entry was reported by Meiyu *et al*. [80]. They indicated that binding of SPMG either to soluble oligomeric rgp120 or to complexed rgp120–sCD4 mainly resided in the V3 loop region. The V3 loop of gp120, considered as a positively charged region, was targeted by negatively charged polysaccharides. In addition, the pre-incubation of SPMG with rgp120 triggered partial suppression of rgp120 binding to sCD4. Thus, they suggested that SPMG either shares common binding sites on gp120 with sCD4 or masks the docking sites of gp120 for sCD4. Finally, SPMG was shown to be less accessible for sCD4 when sCD4 was pre-combined with rgp120, though SPMG multivalently bound to sCD4 with relatively low affinity. However, SPMG may suppress the multivalent binding of rgp120 to sCD4 receptor when SPMG is added either prior to or after the interaction of rgp120 with sCD4. These effective suppressions indicate that SPMG endows both preventive and therapeutic potential on HIV-1 entry.

Fucans are a class of high molecular weight sulfated polysaccharides. They are widely distributed in several species of brown algae and composed of a mainly repeating chain of fucose. The sulfated fucans from the seaweed species *Dictyota mertensii*, *Lobophora variegata*, *Spatoglossum schroederi* and *Fucus vesiculosus* were reported to inhibit HIV reverse transcriptase (RT) by Queiroz *et al*. [84]. They have indicated that the galactofucan fraction from *L. variegate*, which is rich in galactose, fucose and glucose with a lower sulfate content, had a marked inhibitory effect on reverse transcriptase, with 94% inhibition for synthetic polynucleotides at a concentration of 1.0 μg/mL. On the other hand, fucan A from *S. schroederi* and *D. mertensii*, which contains mainly fucose with a lower sulfate level, showed a high inhibitory effect on RT enzyme at 1.0 mg/mL, with 99.03 and 99.3% inhibition, respectively. Meanwhile, fucan B from *S. schroederi*, which contains galactose, fucose and high sulfate level, showed a lower inhibitory activity (53.9%) at the same concentration. Taking another approach, the authors purified a fucan fraction from *F. vesiculosus*, a homofucan containing only sulfated fucose with high sulfate content, which exhibited high inhibitory activity of HIV on RT. This fraction inhibited 98.1% of the reaction with poly(rA)-oligo(dT) at a concentration of 0.5 mg/mL. In addition, they modified SPs by carboxyreduction and desulfation to determine the structure-activity relationship. These modified conditions reduced the inhibitory activities of these polysaccharides for RT approximately four-fold. From these results, they have suggested that fucan activity is dependent on both the ionic changes and the sugar rings that act to spatially orientate the charges in a configuration and recognizes the enzyme, thus determining the specificity of the binding.

In the recent study, Trinchero *et al*. [85] have shown that galactofucan fractions from the brown algae *Adenocystis utricularis* exhibited anti-HIV-1 activity *in vitro*. Among five fractions, EA1-20 and EC2-20 had a strong inhibitory effect on HIV-1 replication *in vitro* with low IC_50_ values (0.6 and 0.9 μg/mL, respectively). Additionally, EA1-20 and EC2-20 displayed this capacity against wild type and drug-resistant HIV-1 strains. For active fractions, it was also shown that the inhibitory effect was not due to an inactivating effect on the viral particles but rather to a blockade of early events of viral replication. Based on these results, seaweed-derived SPs are regarded as good candidates for further studies on prevention of HIV-1 infection.

Beside macroalgae, microalgae are also considered as sources of novel SPs. *Navicula directa* is a diatom often collected at a sluice gate of deep sea water. Naviculan, a sulfated polysaccharide isolated from *N. directa* as a novel antiviral agent, consisted of fucose, xylose, galactose, mannose, rhamnose, and other trace amounts of sugar moieties. Lee *et al*. [82] reported that naviculan possesses antiviral activity, including against herpes simplex viruses type 1 (HSV-1) and herpes simplex viruses type 2 (HSV-2), displaying IC_50_ values of 14 μg/mL and 7.4 μg/mL, respectively. In addition, naviculan also showed an inhibitory effect on the formation of cell–cell fusion between HIV gp160- and CD4-expressing HeLa cells with an IC_50_ value of 53 μg/mL. From these results, it is indicated that this polysaccharide might act as an inhibitor for HSV and HIV-1 infection.

For the first time, Amornrut *et al*. [79] isolated a new type of D-galactan sulfate from clam *Meretrix petechialis* and the anti-HIV activity of this polysaccharide has been evaluated by the inhibition of syncytia formation. Inhibition of νPE 16-induced syncytia formation in CD4 HeLa cells by d-galactan sulfate was 56% at 200 μg/mL and this inhibitory effect is comparable to the positive control dextran sulfate (95% at the same concentration). The inhibition of syncytia formation might be due to the interference of D-galactan sulfate on CD4-gp 120 binding.

Recent studies demonstrated that SPs could be used as a vaginal antiviral formulation without disturbing essential functions of the vaginal epithelial cells and normal bacterial flora. It will be a continuous challenge to select the most promising drug candidates among the wide array of available polysaccharide compounds. The numerous advantages over other classes of antiviral drugs, such as relatively low production costs, broad spectrum of antiviral properties, low cytotoxicity, safety, wide acceptability and novel modes of action, suggest that SPs are promising drug candidates in the near future.

### 2.4. Lectins

Lectins or carbohydrate-binding proteins are found in a variety of different species, ranging from prokaryotes to corals, algae, fungi, plants, invertebrates and vertebrates. They are involved in many biological processes, including host-pathogen interactions, cell-cell communication, induction of apoptosis, cancer metastasis and differentiation, targeting of cells, as well as recognizing and binding carbohydrates. Interestingly, lectins have the potential to block the binding of HIV to target cells, preventing HIV infection and dissemination [87]. Obviously, HIV-1 envelope glycoprotein gp120 is extensively glycosylated and contains approximately 24 potential N-linked glycosylated sites. These glycosylation sites are occupied with glycans that form almost 50% of the gp120 molecular weight. The gp120 glycans represent high-mannose, hybrid and complex classes and serve as ligands for different lectins [88]. The importance of gp120 glycans for the many aspects of HIV-1 infection makes them suitable targets for anti-HIV-1 treatment or prophylaxis [89–91]. There are several types of marine lectins for which anti-HIV activity has been reported (Table 4). Griffithsin (GRFT), a lectin isolated from the red algae *Griffithsia* sp, displayed potent anti-HIV activity [92]. GRFT is a completely novel protein with a molecular weight of 12.7 kDa. It consists of 120 usual amino acids, including one unusual amino acid at position 31 (151 Da). There are no cysteine residues among its 121 amino acids and no homology to any of the proteins or translated nucleotide sequences. Most likely, GRFT molecule is formed as a domain-swapped dimer in solution [92]. Mori *et al*. [92] determined that both native and recombinant GRFT potently inhibited the cytopathic effects of laboratory strains and clinical primary isolates of HIV-1 on T-lymphoblastic cells at concentrations as low as 0.043 μM. It was also shown to be active against both T-cell tropic and macrophage- tropic strains of HIV-1 at the same concentrations. Furthermore, GRFT blocked cell-cell fusion between chronically infected and uninfected cells at sub-nanomolar concentrations. In addition, it aborted the binding of CD4-dependent glycoprotein gp120 to receptor-expressing cells in a glycosylation-dependent manner, and prevent gp120 binding to 2G12 and 48d monoclonal antibody. Interestingly, soluble gp120 binding to GRFT was inhibited by the monosaccharides glucose, mannose and Glc-NAc but not by galactose, xylose, fucose, GalNAc or sialic acid-containing glycoproteins. The Mori *et al*. [92] study also indicated that GRFT may present four carbohydrate-binding domains, separated by three linker sequences Gly-Gly-Ser-Gly-Gly. This organization for this lectin could explain its unusually potent activity due to the possibility of the formation of multivalent bonds between GRFT and oligosaccharides on gp120. These properties make GRFT a promising potential candidate for development as a future pharmaceutical agent.

In recent years, marine invertebrates are attractive as new sources of unusual lectins. CVL is a 30 kDa β-galactose-specific lectin isolated from the marine worm *Chaetopterus variopedatus* [93]. Wang *et al*. [93] indicated that CVL interacts with the hydroxyl group of C-2 and C-4 in the galactose molecule. They also demonstrated that hydroxyl group at C-6 failed to influence binding of the galactose residues of glycoproteins with CVL while the hydroxyl group at C-3 appeared to be very significant for binding glycoproteins with the lectin. Moreover, they investigated the anti-HIV activity of CVL *in vitro*. Results showed that CVL inhibited cytopathic effect induced by HIV-1 and the production of viral p24 antigen at the early stage of virus replication with EC_50_ values of 0.0043 and 0.057 μM, respectively. They also found that CVL could block the cell-to-cell fusion process of HIV infected and uninfected cells with an EC_50_ value of 0.073 μM. Finally, CVL was showed to abort 86% and 21% of HIV-1 entry into host cells at concentrations of 0.33 μM and 0.07 μM, respectively. It is clear that CVL isolated from the marine worm *C. variopedatus* could be a potential candidate to prevent HIV-1 entry into cells.

In addition, the marine worm *Serpula vermicularis* was also subject as a source of new lectins. SVL is a GlcNAc-specific lectin newly purified and characterized by Molchanova *et al*. [94]. This lectin is a homotetrameric glycoprotein in which two identical subunits are connected with disulfide bonds. It is mainly composed of aspartic and glutamic acids, glycine, valine and serine; with relatively lower content of basic amino acids and cysteine. The sequence of N-terminal amino acids of SVL was determined as ADTPCQMLGSRYGWR. SVL was proven to potently inhibit the production of viral p24 antigen and cytopathic effects induced by HIV-1 in host cells at EC_50_ values of 0.23 and 0.15 μg/mL, respectively.

Furthermore, Luk’yanov *et al*. [95] evaluated the anti-HIV activity of lectins isolated from marine invertebrates *Crenomytilus grayanus*, *Didemnum ternatanum* and *Serpula vermicularis*. CGL, a lectin isolated from *C. grayanus* mussel, exhibits a very high affinity to the glycoproteins of mucin type. DTL and DTL-A are lectins isolated from the ascidium *D. ternatanum*. DTL, a GlcNAc-specific lectin with a shorter carbohydrate-binding site, recognizes only terminal residues of GlcNAc and does not recognize the chitobiose core. DTL reveals *N*-chains of hybrid type rather than the carbohydrate chains of complex and highly mannose type. DTL-A, a GlcNAc/GalNAc and heparin-binding lectin, can be used to detect glycoproteins containing α-bound residues of GlcNAc/GalNAc and some SPs due to the peculiarities of its carbohydrate specificity. SVL-1 and SVL-2 are Ca^2+^-independent lectins, revealed in the marine worm *S. vermicularis*. SVL-1 is a mannan-binding lectin with molecular mass of 65 kDa while SVL-2 is a GlcNAc-specific lectin with molecular mass of 50 kDa. Their composition consists of two subunits connected with each other by disulfide bonds forming a tetramer. These lectins were reported to exhibit anti HIV-1 activity in *in vitro* experiments. The DTL, DTL-A, SVL-2, and CGL inhibited the HIV-1 III_B_-induced syncytium formation in C8166 cells with EC_50_ values of 0.002, 0.36, 0.15 and 27.88 μg/mL, respectively. Moreover, these lectins were confirmed to effectively inhibit virus replication and p24 antigen production at EC_50_ values as shown in Table 4. Among these lectins, DTL, DTL-A and CGL displayed activity against cellular fusion between the H9/HIV-1 chronically infected cells and the C8166 uninfected cells at the following concentrations (EC_50_): 1.37, 6.97 and 35.12 μg/mL, respectively. According to their results, DTL may be regarded as a potential candidate for the development of novel antiviral agents.

### 2.5. Bioactive Peptides

Marine-derived bioactive peptides have been isolated widely by enzymatic hydrolysis of marine organisms [96–100]. Proteolytic enzymes derived from microbes, plants and animals can be used for the hydrolysis process of marine proteins to develop bioactive peptides [101]. Bioactive peptides are inactive within the sequence of their parent protein and can be released by enzymatic hydrolysis [102]. Moreover, bioactive peptides usually contain 3–20 amino acid residues, and their activities are based on the amino acid composition and sequence [103]. Marine-derived bioactive peptides have been shown to possess many physiological functions, including anti-hypertensive or angiotensin-I-converting enzyme inhibition [104], anti-oxidant [105,106], anti-coagulant [107,108], and anti-microbial [109,110] activities. Additionally, several studies have reported the anti-HIV activity of marine bioactive peptides (Table 5).

Lee and Maruyama [111] considered that oyster produces antiviral and antibacterial substances for preventing infectious diseases. Thus, they searched for HIV-1 protease-inhibiting substances from oyster *Crassostrea gigas*. They observed and isolated two peptides, Leu-Leu-Glu-Tyr-Ser-Ile (1) and Leu-Leu-Glu-Tyr-Ser-Leu (2), which inhibited HIV-1 protease in thermolysin hydrolysate of oyster protein. The peptide 1 and 2 showed strong inhibition of HIV-1 protease at IC_50_ values of 20 and 15 nM, respectively. Moreover, these peptides behaved as competitive inhibitors for HIV-1 protease with *K*i values of 13 and 10 nM, respectively. Lee and Maruyama [111] confirmed that the presence of C-, N-terminal hydrophobic amino acids and the length of the amino acid sequence in these peptides are important for their inhibitory activity.

Over the years, sponges have been known as a source of novel bioactive peptides. The novel and unique structural features of these peptidic metabolites have generated considerable interest. Cyclic depsipeptides isolated from a number of marine sponges have reported to be active as HIV inhibitors [119]. Callipeltin A, a novel antiviral cyclic depsidecapeptide from sponge of the genus *Callipelta*, contains four amino acids in the L configuration, alanine, leucine, threonine (two residues); one (arginine) in the D configuration; two *N*-methyl amino acids, *N*-methyl alanine and *N*-methyl glutamine; a methoxy tyrosine, a 3,4-dimethyl-L-glutamine; and a 4-amino-7-guanidino-2,3-dihydroxypentanoic acid (AGDHE), formally derived from L-arginine [112]. Callipeltin A exhibited the inhibition of cytopathic effects induced by HIV-1 in CEM4 lymphocytic cell lines at an ED_50_ value of 0.01 μg/mL. The general structure of callipeltin A was identified to be similar to a family of potent antiviral, didemnins, which support that it possesses anti-HIV activity.

Likewise, Ford *et al*. [113] have isolated novel cyclic depsipeptides papuamides A and B from sponges *Theonella mirabilis* and *Theonella swinhoei*. These peptides contain not only unusual amino acids, including 3,4-dimethylglutamine, *β*-methoxytyrosine, 3-methoxyalanine, and 2,3-diaminobutanoic acid or 2-amino-2-butenoic acid residues, but also the first marine-derived peptides reported to contain 3-hydroxyleucine and homoproline residues. These peptides also contain a previously undescribed 2,3-dihydroxy-2,6,8-trimethyldeca-(4*Z*,6*E*)-dienoic acid moiety N-linked to a terminal glycine residue. Papuamides A and B were reported to block the infection of human T-lymphoblastoid cells by HIV-1 sub(RF) *in vitro* with an EC_50_ of approximately 4 ng/mL. The inhibitory activity of papuamides A was due to blockage by this peptide at the initial stage of the viral life cycle, but was not HIV-1 envelope glycoprotein specific [120]. At a similar concentration to papuamide A, papuamide B also prevents viral entry via interaction of this peptide with phospholipid present on the viral membrane [120]. Another anti-HIV candidate from the sponge is the microspinosamide, a new cyclic depsipeptide isolated from *Sidonops microspinosa*. This peptide incorporates 13 amino acid residues, and it is the first naturally occurring peptide to contain a *β*-hydroxy-*p* bromophenylalanine residue [114]. Rashid *et al*. [114] demonstrated that microspinosamide is capable of inhibiting the cytopathic effect of HIV-1 infection in an XTT-based *in vitro* assay with an EC_50_ value of approximately 0.2 μg/mL.

In another study, Oku *et al*. [115] isolated a new HIV-inhibitory depsipeptide, neamphamide A, from the marine sponge *Neamphius huxleyi*. Neamphamide A contains an amino acid sequence of l-Leu, l-*N*MeGln, d-Arg, d- and l-Asn, two residues of d-*allo*-Thr, l-homoproline, (3*S,*4*R*)-3,4-dimethyl- lglutamine, *β*-methoxytyrosine, 4-amino-7-guanidino-2,3-dihydroxyheptanoic acid and an amide-linked 3-hydroxy-2,4,6-trimethylheptanoic acid moiety. Neamphamide A was reported to have cytoprotective activity against HIV-1 infection (EC_50_, 28 nM). Similar to neamphamide A, mirabamides obtained from the marine sponge *Siliquariaspongia mirabilis* also potently inhibit HIV-1 fusion [116]. Among mirabamides, mirabamide A was found to be powerful against HIV-1 in neutralization and fusion assays with respective IC_50_ values of 40 and 140 nM, while mirabamides C and D were shown to be less effective (IC_50_ values between 140 nM and 1.3 *μ*M for mirabamide C and 190 nM and 3.9 μM for mirabamide D). Further, Plaza *et al*. [116] confirmed that mirabamides inhibit HIV-1 at the level of membrane fusion, presumably through interactions with HIV-1 envelope glycoproteins. Other novel peptides, celebesides A and theopapuamide B, were also isolated from sponges of the same previously mentioned *S. mirabilis* [117]. Celebesides A is cyclic depsipeptide that incorporates a polyketide moiety and five amino acid residues, among which are the unusual amino acids phosphoserine and 3-carbamoyl threonine. Theopapuamide B, a undecapeptide, comprise two previously unreported amino acids, 3-acetamido-2-aminopropanoic acid and 4-amino-2,3-dihydroxy-5-methylhexanoic acid. Celebesides A displayed an inhibition of HIV-1 entry with an IC_50_ value of 1.9 μg/mL, while theopapuamide B was active in the neutralization assay with an IC_50_ value of 0.8 μg/mL. Plaza *et al*. [117] indicated that the inhibitory activity of Celebesides A is due to the presence of a phosphoserine residue conserved in the above-mentioned anti-HIV peptides but absent in the inactive theopapuamide. However, this hypothesis was ruled out by evidence given in the study of Zampella and collaborators [118]. Zampella *et al*. [118] isolated a new anti-HIV cyclodepsipeptide, homophymine A, from the marine sponge *Homophymia* sp. This peptide contains an amide-linked 3-hydroxy-2,4,6-trimethyloctanoic acid moiety and 11 amino acid residues, including four unusual amino acid residues: (2*S*,3*S*,4*R*)-3,4-diMe-Gln, (2*R*,3*R*,4*S*)-4-amino-2,3-dihydroxy-1,7-heptandioic acid, l-ThrOMe, and (2*R*,3*R*,4*R*)-2-amino-3-hydroxy-4,5-dimethylhexanoic acid. Obviously, homophymine A lacks *β-*methoxytyrosine residue which is replaced by an *O*-methyl threonine residue. Nevertheless, homophymine A was reported to potentially exhibit cytoprotective activity against HIV-1 infection with an IC_50_ value of 75 nM. The antiviral activity found in homophymine A ruled out the hypothesis that β-methoxytyrosine is essential for antiviral activity.

## 3. Prospects of Marine Anti-HIV Drugs

HIV-caused AIDS is a major health problem worldwide, especially in developing countries. The discovery of medicinal agents specifically capable of inhibiting HIV is urgently required to prevent globally widespread infection. Natural products derived from marine organisms are excellent sources for the effective discovery of anti-HIV agents. However, the development of marine drugs still faces several challenges, such as toxic side effects, large-scale production and cultivation, and drug resistance. As mentioned above, sponge-derived compounds were shown to exhibit strong inhibition of HIV entry but have toxic effects, which is an obstacle for development as commercial drugs. However, it is possible to solve this problem through the application of biochemical technologies. These techniques will allow the manipulation of naturally occurring compounds, such as removal or modification of toxic groups present in the compound to produce chemical derivatives that are far superior to the original [121]. Thus, this application will allow the production of compounds with reduced cytotoxicities and increased specificities.

Another important challenge for HIV treatment is drug resistance. Nevertheless, a virus that has developed resistance to a particular drug may not be resistant to other naturally occurring derivatives, which display similar antiviral activities [122]. Therefore, the different derivatives of a common class compound that are synthesized by multiple organisms may be a solution for new drugs discovery. Furthermore, the numerous undiscovered unique metabolites in the marine environment are interesting sources to increase numbers of novel drugs against otherwise drug-resistant HIV strains.

In addition, the anti-HIV compounds produced by marine organisms are often found in low natural abundance. Thus, it is really difficult to make a large-scale production of these compounds. One strategy to increase the yields of natural products consists of the identification, cloning and expression of genes associated with biosynthetic machineries and subsequent production of the compounds enzymatically in a heterologous host. This genetic approach also allows the production of novel metabolites via introducing novel biosynthesis genes into microorganisms, which will result in the synthesis of novel metabolites.

## 4. Conclusion

Recent studies have provided evidence that marine-derived anti-HIV agents may play a vital role against HIV. The possibilities of designing new drug candidates and pharmaceuticals to support reducing or regulating HIV infection related chronic malfunctions are promising. Moreover, these evidences suggest that due to valuable biological functions with beneficial health effects, marine-derived anti-HIV agents have potential as active ingredients for preparation of novel pharmaceutical products. Until now, most of studies on anti-HIV activity of marine-derived HIV inhibitors have been observed *in vitro* or in mouse model systems. Therefore, further research studies are needed in order to investigate their activities in human subjects.

## Figures and Tables

**Table 1:** Inhibitory effects of phlorotannins on the HIV-1 reverse transcriptase (RT), integrase and protease.

Compound	IC_50_^a^ (μM)			Ref.
	RT	Integrase	Protease	
8,8′-bieckol	0.51		81.5	[24]
8,4‴-dieckol	5.31		36.9	[24]
diphlorethohydroxycarmalol	9.1	25.2		[25]
6,6′-bieckol	1.07			[26]

a 50% inhibitory concentration.

**Table 2:** Anti-HIV-1 activity of chitin sulfates *in vitro* [42].

Compound	Mw (kDa)	EC_50_^a^ (μg/mL)	CC_50_^b^ (μg/mL)
C-6S	58	57.0	>1000
C-3S	28	9.6	>1000
C-2,3S	16	0.28	>1000
Curdlan sulfate	79	0.10	>1000

a 50% effective concentration.

b 50% cytotoxic concentration.

**Table 3:** Marine SPs-derived anti-HIV agents.

SPs	Major units	Sources	Ref.
Sulfated glucuronogalactan	Galactose	Red algae *Schizymenia dubyi*	[77]
Kakelokelose	Mannose	Pacific tunicate *Didemnum molle*	[78]
Sulfated β-galactan	Galactose	Clam *Meretrix petechialis*	[79]
Sulfated polymannoroguluronate	Mannuronate	Brown algae	[80]
Sulfated polymannuronate	Mannuronate	Brown algae	[81]
Naviculan	Fucose, Xylose, Galactose, Mannose, Rhamnose	Diatom *Navicula directa*	[82]
Sulfated galactans	Galactose, Xylose	Red algae *Grateloupia filicina, Grateloupia longifolia*	[83]
Sulfated fucans	Fucose	Brown algae *Dictyota mertensii*, *Lobophora variegate, Fucus vesiculosus, Spatoglossum schröederi*	[84]
Galactofucan	Fucose, Galactose	Brown algae *Adenocystis utricularis*	[85]

**Table 4:** Anti-HIV activity of marine lectins.

Species	Lectin	Carbohydrate specificity	Activity	EC_50_^a^	Ref.

Red algae *Griffithsia* sp.	*GRFT*	Man/Glc-specific lectin	Against T cell tropic and macrophage tropic strains of HIV-1	Ranging from 0.043–0.63 μM	[92]
			Abort cell-to-cell fusion and transmission of HIV-1 infection	Ranging from 0.043–0.63 μM	

Marine worm *Chaetopterus variopedatus*	CVL	β-galactose-specific lectin	Inhibit HIV-induced syncytium formation	0.0043 μM	[93]
			Inhibit HIV-1 p24 production	0.057 μM	

Marine worm *Serpula vermicularis*	SVL	GlcNAc-specific lectin	Inhibit HIV-induced syncytium formation	0.15 μg/mL	[94]
			Inhibit HIV-1 p24 production	0.23 μg/mL	

Marine mussel *Crenomytilus grayanus*	CGL	High affinity to the glycoproteins of mucin type	Inhibit HIV-replication	45.7 μg/mL	[95]

Ascidium *Didemnum ternatanum*	DTL	GlcNAc-specific lectin	Inhibit HIV-replication	0.006 μg/mL	[95]

Ascidium *Didemnum ternatanum*	DTL-A	GlcNAc/GalNAc and heparin-binding lectin	Inhibit HIV-replication	0.59 μg/mL	[95]

Marine worm *Serpula vermicularis*	SVL-1	Mannan-binding lectin	Inhibit HIV-replication	89.1 μg/mL	[95]

Marine worm *Serpula vermicularis*	SVL-2	GlcNAc-specific lectin	Inhibit HIV-replication	0.23 μg/mL	[95]

a 50% effective concentration.

**Table 5:** HIV-1 inhibitory effect of marine peptides.

Sources	Peptide name	Activity	Potency	Ref.
Oyster *Crassostrea gigas*	Peptide 1 Peptide 2	Inhibit HIV-1 protease	IC_50_: 20 nM (1) 15 nM (2)	[111]
Marine sponge *Callipelta*	Callipeltin A	Inhibit cytopathic effects induced by HIV-1	EC_50_: 0.01 μg/mL	[112]
Marine sponge *Theonella mirabilis* *Theonella swinhoei*	Papuamides A Papuamides B	Inhibit HIV-1 infection	EC_50_: 4 ng/mL	[113]
Marine sponge *Sidonops microspinosa*	Microspinosamide	Inhibit cytopathic effect of HIV-1 infection	EC_50_: 0.2 μg/mL	[114]
Marine sponge *Neamphius huxleyi*	Neamphamide A	Against HIV-1 infection	EC_50_: 28 nM	[115]
Marine sponge *Siliquariaspongia mirabilis*	Mirabamide A Mirabamide C Mirabamide D	Inhibit HIV-1 neutralization and fusion	IC_50_: 0.04 and 0.14 μM (A) 0.14 and 1.3 μM (C) 0.19 and 3.9 μM (D)	[116]
Marine sponge *Siliquariaspongia mirabilis*	Celebesides A Theopapuamide B	Block HIV-1 entry (A) Neutralize HIV-1 (B)	IC_50_: 1.9 μg/mL (A) 0.8 μg/mL (B)	[117]
Marine sponge *Homophymia* sp	Homophymine A	Against HIV-1 infection	IC_50_: 75 nM	[118]

EC_50_: 50% effective concentration.

IC_50_: 50% inhibitory concentration.

## Abbreviations

AIDS: acquired immune deficiency syndrome
SCOS: sulfated chitooligosaccharide
SPs: sulfated polysaccharides

## References

[1] Ojewole E Mackraj I Naidoo P Govender T Exploring the use of novel drug delivery systems for antiretroviral drugs Eur J Pharm Biopharm 2008 70 697 710 18655830 10.1016/j.ejpb.2008.06.020

[2] Govender T Ojewole E Naidoo P Polymeric nanoparticles for enhancing antiretroviral drug therapy Drug Deliv 2008 15 493 501 18720133 10.1080/10717540802321776

[3] Clavel F Hance AJ HIV drug resistance N Engl J Med 2004 350 1023 1035 14999114 10.1056/NEJMra025195

[4] Lee SA Hong SK Suh CI Oh MH Park JH Choi BW Park SW Paik SY Anti-HIV-1 efficacy of extracts from medicinal plants J Microbiol 2010 48 249 252 20437159 10.1007/s12275-009-0176-9

[5] Tantillo C Ding J Jacobo-Molina A Nanni RG Boyer PL Hughes SH Pauwels R Andries K Janssen PAJ Arnold E Locations of anti-AIDS drug binding sites and resistance mutations in the three-dimensional structure of HIV-1 reverse transcriptase. Implications for mechanisms of drug inhibition and resistance J Mol Biol 1994 243 369 387 7525966 10.1006/jmbi.1994.1665

[6] Lipsky JJ Antiretroviral drugs for AIDS Lancet 1996 348 800 803 8813990 10.1016/S0140-6736(95)12333-4

[7] Tzileleka LA Vagias C Roussis V Natural products with anti-HIV activity from marine organisms Curr Top Med Chem 2003 3 1512 1535 14529524 10.2174/1568026033451790

[8] Singh IP Bharate SB Bhutani KK Anti-HIV natural products Curr Sci 2005 89 269 290

[9] Schaeffer DJ Krylov VS Anti-HIV activity of extracts and compounds from algae and cyanobacteria Ecotox Environ Safe 2000 45 208 227 10.1006/eesa.1999.1862 10702339

[10] Vlietinck AJ De Bruyne T Apers S Pieters LA Plantderived leading compounds for chemotherapy of human immunodeficiency virus (HIV) infection Planta Med 1998 64 97 109 9525100 10.1055/s-2006-957384

[11] De Clercq E Current lead natural products for the chemotherapy of human immuno deficiency virus infection Med Res Rev 2000 20 323 349 10934347 10.1002/1098-1128(200009)20:5<323::aid-med1>3.0.co;2-a

[12] Kong JM Goh NK Chia LS Chia TF Recent advances in traditional plant drugs and orchids Acta Pharmacol Sin 2003 24 7 21 12511224

[13] Wang RR Gu Q Wang YH Zhang XM Yang LM Zhou J Chen JJ Zheng YT Anti-HIV-1 activities of compounds isolated from the medicinal plant *Rhus chinensis* J Ethnopharmacol 2008 17 249 256 18343612 10.1016/j.jep.2008.01.037

[14] Hupfeld J Efferth T Drug resistance of human immunodeficiency virus and overcoming it by natural products In Vivo 2009 23 1 6 19368117

[15] Aneiros A Garateix A Bioactive peptides from marine sources: pharmacological properties and isolation procedures J Chromatogr B Analyt Technol Biomed Life Sci 2004 803 41 53 10.1016/j.jchromb.2003.11.005 15025997

[16] Wijesekara I Kim SK Angiotensin-I-converting enzyme (ACE) inhibitors from marine resources: prospects in the pharmaceutical industry Mar Drugs 2010 8 1080 1093 20479968 10.3390/md8041080 PMC2866476

[17] Pomponi SA The oceans and human health: the discovery and development of marine-derived drugs Oceonogrophy 2001 14 78 87

[18] Targett NM Arnold TM Predicting the effects of brown algal phlorotannins on marine herbivores in tropical and temperate oceans J Phycol 1998 34 195 205

[19] Shibata T Kawaguchi S Hama Y Inagaki M Yamaguchi K Nakamura T Local and chemical distribution of phlorotannins in brown algae J Appl Phycol 2004 16 291 296

[20] Ahn GN Kim KN Cha SH Song CB Lee J Heo MS Yeo IK Lee NH Jee YH Kim JS Heu MS Jeon YJ Antioxidant activities of phlorotannins purified from *Ecklonia cava* on free radical scavenging using ESR and H_2_O_2_-mediated DNA damage Eur Food Res Technol 2007 226 71 79

[21] Singh IP Bharate SB Phloroglucinol compounds of natural origin Nat Prod Rep 2006 23 558 591 16874390 10.1039/b600518g

[22] Glombitza KW Li SM Hydroxyphlorethols from the brown alga *Carpophyllum maschalocarpum* Phytochemistry 1991 30 2741 2745

[23] La Barre S Potin P Leblanc C Delage L The halogenated metabolism of brown algae (Phaeophyta), its biological importance and its environmental significance Mar Drugs 2010 8 988 1010 20479964 10.3390/md8040988 PMC2866472

[24] Ahn MJ Yoon KD Min SY Lee JS Kim JH Kim TG Kim SH Kim NG Huh H Kim J Inhibition of HIV-1 reverse transcriptase and protease by phlorotannins from the brown alga Ecklonia cava Biol Pharm Bull 2004 27 544 547 15056863 10.1248/bpb.27.544

[25] Ahn MJ Yoon KD Kim CY Kim JH Shin CG Kim J Inhibitory activity on HIV-1 reverse transcriptase and integrase of a carmalol derivative from a brown alga *Ishige okamurae* Phytother Res 2006 20 711 713 16775811 10.1002/ptr.1939

[26] Artan M Li Y Karadeniz F Lee SH Kim MM Kim SK Anti-HIV-1 activity of phloroglucinol derivative, 6,6-bieckol, from Eckloniacava Bioorg Med Chem 2008 16 7921 7926 10.1016/j.bmc.2008.07.078 18693022

[27] Carroll SS Olsen DB Bennett CD Gotlib L Graham DJ Condraq JH Stern AM Shafer JA Kuo LC Inhibition of HIV-I reverse transcriptase by pyridinone derivatives J Biol Chem 1993 268 276 281 7677997

[28] Zhang H Vrang L Backbro K Lind P Sahlberg C Unge T Oberg B Inhibition of human immunodeficiency virus type 1 wild-type and mutant reverse transcriptases by the phenyl ethyl thiazolyl thiourea derivatives trovirdine and MSC-127 Antivir Res 1995 28 331 342 8669892 10.1016/0166-3542(95)00056-9

[29] Ngo DN Kim MM Kim SK Chitin oligosaccharides inhibit oxidative stress in live cells Carbohydr Polym 2008 74 228 234

[30] Shahidi F Arachchi JKV Jeon YJ Food applications of chitin and chitosans Trends Food Sci Technol 1999 10 37 51

[31] Kim SK Nghiep ND Rajapakse N Therapeutic prospectives of chitin, chitosan and their derivatives J Chitin Chitosan 2006 11 1 10

[32] Jayakumar R New N Nagagama H Furuike T Tamura H Synthesis, characterization and biospecific degradation behavior of sulfated chitin Macromol Symp 2008 264 163 167

[33] Suwan J Zhang Z Li B Vongchan P Meepowpan P Zhang F Mousa SA Mousa S Premanode B Kongtawelert P Linhardt RJ Sulfonation of papain-treated chitosan and its mechanism for anticoagulant activity Carbohydr Res 2009 344 1190 1196 19476923 10.1016/j.carres.2009.04.016 PMC2727590

[34] He Q Ao Q Wang A Gong Y Zhao N Zhang X *In vitro* cytotoxicity and protein drug release properties of chitosan/heparin microspheres Tsinghua Sci Technol 2007 12 361 365

[35] Thierry B Merhi Y Silver J Tabrizian M Biodegradable membrane-covered stent from chitosan-based polymers J Biomed Mater Res A 2005 75 556 566 16094632 10.1002/jbm.a.30450

[36] Prabaharan M Reis RL Mano JF Carboxymethyl chitosan-graft-phosphatidylethanolamine: Amphiphilic matrices for controlled drug delivery React Funct Polym 2007 67 43 52

[37] Jayakumar R Nwe N Tokura S Tamura H Sulfated chitin and chitosan as novel biomaterials Int J Biol Macromol 2007 40 175 181 16893564 10.1016/j.ijbiomac.2006.06.021

[38] Saiki I Murata J Nakajima M Tokura S Azuma I Inhibition by sulfated chitin derivatives of invasion through extracellular matrix and enzymatic degradation by metastatic melanoma cells Cancer Res 1990 50 3631 3637 2340512

[39] Vasyukova NI Chalenko GI Gerasimova NG Perekhod EA Ozeretskovskaya OL Irina AV Varlamov VP Albulov AI Chitin and chitosan derivatives as elicitors of potato resistance to late blight Appl Biochem Micro 2000 36 372 376 10994192

[40] Xing R Liu S Yu H Zhang Q Li Z Li P Preparation of low-molecular-weight and highsulfate-content chitosans under microwave radiation and their potential antioxidant activity *in vitro* Carbohyd Res 2004 339 2515 2519 10.1016/j.carres.2004.08.013 15476712

[41] Sosa MA Fazely F Koch JA Vercellotti SV Ruprecht RM *N*-carboxymethylchitosan-*N,**O*-sulfate as an anti-HIV-1 agent Biochem Biophys Res Commun 1991 174 489 496 1704225 10.1016/0006-291x(91)91443-g

[42] Nishimura SI Kai H Shinada K Yoshida T Tokura S Kurita K Nakashima H Yamamoto N Uryu T Regioselective syntheses of sulfated polysaccharides: specific anti-HIV-1 activity of novel chitin sulfates Carbohydr Res 1998 306 427 433 9648250 10.1016/s0008-6215(97)10081-7

[43] Lee DS Kim YM Lee MS Ahn CB Jung WK Je JY Synergistic effects between aminoethyl-chitosans and β-lactams against methicillin-resistant Staphylococcus aureus (MRSA) Bioorg Med Chem Lett 2010 20 975 978 20036533 10.1016/j.bmcl.2009.12.049

[44] Artan M Karadeniz F Kim MM Kim SK Chitosan derivatives as HIV-1 inhibitors J Biotechnol 2008 136S S527 540

[45] Dou JL Tan CY Du YG Bai XF Wang KY Ma XJ Effects of chitooligosaccharides on rabbit neutrophils *in vitro* Carbohydr Polym 2007 69 209 213

[46] Jeon YJ Kim SK Continuous production of chitooligosaccharides using a dual reactor system Process Biochem 2000 35 623 632

[47] Jeon YJ Kim SK Production of chitooligosaccharides using ultrafiltration membrane reactor and their antibacterial activity Carbohydr Polym 2000 41 133 141

[48] Yang EJ Kim JG Kim JY Kim SC Lee NH Hyun CG Anti-inflammatory effect of chitosan oligosaccharides in RAW 264.7 cells Cent Eur J Biol 2010 5 95 102

[49] Chae SY Jang MK Nah JW Influence of molecular weight on oral absorption of water soluble chitosans J Control Release 2005 102 383 394 15653159 10.1016/j.jconrel.2004.10.012

[50] Hong SP Kim MH Oh SW Han CH Kim YH ACE inhibitory and antihypertensive effect of chitosan oligosaccharides in SHR Korean J Food Sci Technol 1998 30 1476 1479

[51] Park PJ Je JY Kim SK Free radical scavenging activity of chitooligosaccharides by electron spin resonance spectrometry J Agric Food Chem 2003 51 4624 4627 14705887 10.1021/jf034039+

[52] Park PJ Lee HK Kim SK Preparation of hetero-chitooligosaccharides and their antimicrobial activity on *Vibrio parahaemolyticus* J Microbiol Biotechnol 2004 14 41 47

[53] Shen KT Chen MH Chan HY Jeng JH Wang YJ Inhibitory effects of chitooligosaccharides on tumor growth and metastasis Food Chem Toxicol 2009 47 1864 1871 19427889 10.1016/j.fct.2009.04.044

[54] Jeon YJ Kim SK Antitumor activity of chitosan oligosaccharides produced in ultrafiltration membrane reactor system J Microbiol Biotechnol 2002 12 503 507

[55] Jeon YJ Kim SK Potential immuno-stimulating effect of antitumoral fraction of chitosan oligosaccharides J Chitin Chitosan 2001 6 163 167

[56] Liu B Liu WS Han BQ Sun YY Antidibetic effects of chito-oligosaccharides on pancreatic islet cells in streptozotocin-induced diabetic rats World J Gastroenterol 2007 13 725 731 17278195 10.3748/wjg.v13.i5.725 PMC4066005

[57] Kim KN Joo ES Kim KI Kim SK Yang HP Jeon YJ Effect of chitosan oligosaccharides on cholesterol level and antioxidant enzyme activities in hypercholesterolemic rat J Korean Soc Food Sci Nutr 2005 34 36 41

[58] Miura T Usami M Tsuura Y Ishida H Seino Y Hypoglycemic and hypolipidemic effect of chitosan in normal and neonatal streptozotocin-induced diabetic mice Biol Pharm Bull 1995 18 1623 1625 8593495 10.1248/bpb.18.1623

[59] Yoon NY Ngo DN Kim SK Acetylcholinesterase inhibitory activity of novel chitooligosaccharide derivatives Carbohydr Polym 2009 78 869 872

[60] Park PJ Je JY Jung WK Kim SK Anticoagulant activity of heterochitosans and their oligosaccharide sulfates Eur Food Res Technol 2004 219 529 533

[61] Cho EJ Rahman A Kim SW Baek YM Hwang HJ Oh JY Hwang HS Lee SK Yun JW Chitosan oligosaccharides inhibit adipogenesis in 3T3-L1 adipocytes J Microbiol Biotechnol 2008 18 80 87 18239421

[62] Artan M Karadeniz F Karagozlu MZ Kim MM Kim SK Anti-HIV-1 activity of low molecular weight sulfated chitooligosaccharides Carbohydr Res 2010 345 656 662 20117763 10.1016/j.carres.2009.12.017

[63] Vijayavel K Anbuselvam C Balasubramanian MP Free radical scavenging activity of the marine mangrove Rhizophora apiculata bark extract with reference to naphthalene induced mitochondrial dysfunction Chem Biol Interact 2006 163 170 175 16860784 10.1016/j.cbi.2006.06.003

[64] Alban S Franz G Partial synthetic glucan sulfates as potential new antithrombotics: a review Biomacromolecules 2001 2 354 361 11749192 10.1021/bm010032u

[65] Costa LS Fidelis GP Cordeiro SL Oliveira RM Sabry DA Camara RBG Nobre LTDB Costa MSSP Almeida-Lima J Farias EHC Leite EL Rocha HAO Biological activities of sulfated polysaccharides from tropical seaweeds Biomed Pharmacother 2010 64 21 28 19766438 10.1016/j.biopha.2009.03.005

[66] Ley K Cerrito M Arfors KE Sulfated polysaccharides inhibit leukocyte rolling in rabbit mesentery venules Am J Physiol Heart Circ Physiol 1991 260 H1667 1673 10.1152/ajpheart.1991.260.5.H1667 2035685

[67] Majdoub H Ben Mansour M Chaubet F Roudesli MS Maaroufi RM Anticoagulant activity of a sulfated polysaccharide from the green alga *Arthrospira platensis* Biochim Biophys Acta 2009 1790 1377 1381 19632306 10.1016/j.bbagen.2009.07.013

[68] Synytsya A Kim WJ Kim SM Pohl R Synytsya A Kvasnicka F Copikova J Park YI Structure and antitumor activity of fucoidan isolated from sporophyll of Korean seaweed *Undaria pinnatifida* Carbohydr Polym 2010 81 41 48

[69] Na YS Kim WJ Kim SM Park JW Lee SM Kim SO Synytsya A Park YI Purification, characterization and immunostimulating activity of water-soluble polysaccharide isolated from *Capsosiphon fulvescens* Int Immunopharmacol 2010 10 364 370 20074671 10.1016/j.intimp.2009.12.011

[70] Witvrouw M De Clercq E Sulfated polysaccharides extracted from sea algae as potential antiviral drugs Gen Pharmacol 1997 29 497 511 9352294 10.1016/s0306-3623(96)00563-0

[71] Adhikari U Mateu CG Chattopadhyay K Pujol CA Damonte EB Ray B Structure and antiviral activity of sulfated fucans from *Stoechospermum marginatum* Phytochemistry 2006 67 2474 2482 17067880 10.1016/j.phytochem.2006.05.024

[72] Damonte EB Matulewicz MC Cerezo AS Sulfated seaweed polysaccharides as antiviral agents Curr Med Chem 2004 11 2399 2419 15379705 10.2174/0929867043364504

[73] Luscher-Mattil M Polyanions–a lost chance in the fight against HIV and other virus diseases Antivir Chem Chemoth 2000 11 249 259 10.1177/095632020001100401 10950387

[74] Chattopadhyay N Ghosh T Sinha S Chattopadhyay K Karmakar P Ray B Polysaccharides from *Turbinaria conoides*: Structural features and antioxidant capacity Food Chem 2010 118 823 829

[75] Mandal P Mateu CG Chattopadhyay K Pujol CA Damonte EB Ray B Structural features and antiviral activity of sulfated fucans from the brown seaweed *Cystoseira indica* Antivir Chem Chemother 2007 18 153 162 17626599 10.1177/095632020701800305

[76] Mandal P Pujol CA Carlucci MJ Chattopadhyay K Damonte EB Ray B Sulfated xylomannan of *Scinaia hetai*: Isolation, structural features and antiviral activity Phytochemistry 2008 69 2193 2199 18572208 10.1016/j.phytochem.2008.05.004

[77] Bourgougnon N Lahaye M Quemener B Chermann JC Rimbert M Cormaci M Furnari G Komprobst JM Annual variation in composition and *in vitro* anti-HIV-1 activity of the sulfated glucuronogalactan from *Schizymenia dubyi* (Rhodophyta, Gigartinales) J App Phycol 1996 8 155 161

[78] Riccio R Kinnel RB Bifulco G Scheuer PJ Kakelokelose, a sulfated mannose polysaccharide with anti-HIV activity from the pacific tunicate *Didemnum molle* Tetrahedron Lett 1996 37 1979 1982

[79] Amornrut C Toida T Imanari T Woo ER Park H Linhardt R Wu SJ Kim YS A new sulfated β-galactan from clams with anti-HIV activity Carbohyd Res 1999 321 121 127 10.1016/s0008-6215(99)00188-3 10612006

[80] Meiyu G Fuchuan L Xianliang X Jing L Zuowei Y Huashi G The potential molecular targets of marine sulfated polymannuroguluronate interfering with HIV-1 entry. Interaction between SPMG and HIV-1 rgp120 and CD4 molecule Antivir Res 2003 59 127 135 12895696 10.1016/s0166-3542(03)00068-8

[81] Liu H Geng M Xin X Li F Zhang Z Li J Ding J Multiple and multivalent interactions of novel anti-AIDS drug candidates, sulfated polymannuronate (SPMG)-derived oligosaccharides, with gp120 and their anti-HIV activities Glycobiology 2005 15 501 510 15616125 10.1093/glycob/cwi031

[82] Lee JB Hayashi K Hirata M Kuroda E Suzuki E Kubo Y Hayashi T Antiviral sulfated polysaccharide from *Navicula directa, *a diatom collected from deep-sea water in Toyama bay Biol Pharm Bull 2006 29 2135 2139 17015966 10.1248/bpb.29.2135

[83] Wang SC Bligh SWA Shi SS Wang ZT Hu ZB Crowder J Branford-White C Vella C Structural features and anti-HIV-1 activity of novel polysaccharides from red algae *Grateloupia longifolia* and *Grateloupia filicina* Int J Biol Macromol 2007 41 369 375 17602734 10.1016/j.ijbiomac.2007.05.008

[84] Queiroz KCS Medeiros VP Queiroz LS Abreu LRD Rocha HAO Ferreira CV Juca MB Aoyama H Leite EL Inhibition of reverse transcriptase activity of HIV by polysaccharides of brown algae Biomed Pharmacother 2008 62 303 307 18455359 10.1016/j.biopha.2008.03.006

[85] Trinchero J Ponce NMA Cordoba OL Flores ML Pampuro S Stortz CA Salomon H Turk G Antiretroviral activity of fucoidans extracted from the brown seaweed *Adenocystis utricularis* Phytother Res 2009 23 707 712 19107862 10.1002/ptr.2723

[86] Miao B Li J Fu X Gan L Xin X Geng M Sulfated polymannuroguluronate, a novel anti-AIDS drug candidate, inhibits T cell apoptosis by combating oxidative damage of mitochondria Mol Pharmacol 2005 68 1716 1727 16141310 10.1124/mol.105.015412

[87] Sato T Hori K Cloning, expression, and characterization of a novel anti-HIV lectin from the cultured cyanobacterium *Oscillatoria agardhii* Fish Sci 2009 75 743 753

[88] Geyer H Holschbach C Hunsmann G Schneider J Carbohydrates of human immunodeficiency virus. Structures of oligosaccharides linked to the envelope glycoprotein 120 J Biol Chem 1988 263 11760 11767 2841333

[89] Balzarini J Carbohydrate-binding agents: a potential future cornerstone for the chemotherapy of enveloped viruses Antivir Chem Chemother 2007 18 1 11 17354647 10.1177/095632020701800101

[90] Balzarini J Targeting the glycans of glycoproteins: a novel paradigm for antiviral therapy Nat Rev Microbiol 2007 5 583 597 17632570 10.1038/nrmicro1707 PMC7098186

[91] Balzarini J Laethem KV Daelemans D Hatse S Bugatti A Rusnati M Igarashi Y Oki T Schols D Pradimicin A, a carbohydrate-binding nonpeptidic lead compound for treatment of infections with viruses with highly glycosylated envelopes, such as human immunodeficiency virus J Virol 2007 81 362 373 17050611 10.1128/JVI.01404-06 PMC1797273

[92] Mori T O’Keefe BR Sowder RC II Bringans S Gardella R Berg S Cochran P Turpin JA Buckheit RW Jr McMahon JB Boyd MR Isolation and characterization of Griffithsin, a novel HIV-inactivating protein, from the red alga *Griffithsia* sp J Biol Chem 2005 280 9345 9353 15613479 10.1074/jbc.M411122200

[93] Wang JH Kong J Li W Molchanova V Chikalovets I Belogortseva N Luk’yanov P Zheng YT A β-galactose-specific lectin isolated from the marine worm *Chaetopterus variopedatus* possesses anti-HIV-1 activity Comp Biochem Physiol 2006 142 111 117 10.1016/j.cbpc.2005.10.019 16316787

[94] Molchanova V Chikalovets I Chernikov O Belogortseva N Li W Wang JH Ou Yang DY Zheng YT Lukyanov P A new lectin from the sea worm *Serpula vermicularis*: Isolation, characterization and anti-HIV activity Comp Biochem Physiol 2007 145 184 193 10.1016/j.cbpc.2006.11.012 17258940

[95] Luk’yanov PA Chernikov OV Kobelev SS Chikalovets IV Molchanova VI Li W Carbohydrate-Binding Proteins of Marine Invertebrates Russ J Bioorg Chem 2007 33 161 169 17375673

[96] Je JY Qian ZJ Lee SH Byun HG Kim SK Purification and antioxidant properties of bigeye tuna (*Thunnus obesus*) dark muscle peptide on free radical-mediated oxidation systems J Med Food 2008 11 629 637 19053853 10.1089/jmf.2007.0114

[97] Sheih IC Fang TJ Wu TK Isolation and characterization of a novel angiotensin I-converting enzyme (ACE) inhibitory peptide from the algae protein waste Food Chem 2009 115 279 284

[98] Slizyte R Mozuraityte R Martinez-Alvarez O Falch E Fouchereau-Peron M Rustad T Functional, bioactive and antioxidative properties of hydrolysates obtained from cod (*Gadus morhua*) backbones Process Biochem 2009 44 668 677

[99] Suetsuna K Chen JR Identification of antihypertensive peptides from peptic digest of two microalgae, *Chlorella vulgaris* and *Spirulina platensis* Mar Biotechnol 2001 3 305 309 14961345 10.1007/s10126-001-0012-7

[100] Zhao Y Li B Liu Z Dong S Zhao X Zeng M Antihypertensive effect and purification of an ACE inhibitory peptide from sea cucumber gelatin hydrolysate Process Biochem 2007 42 1586 1591

[101] Simpson BK Nayeri G Yaylayan V Ashie INA Enzymatic hydrolysis of shrimp meat Food Chem 1998 61 131 138

[102] Korhonen H Pihlanto A Bioactive peptides: Production and functionality Int Dairy J 2006 16 945 960

[103] Pihlanto-Leppala A Bioactive peptides derived from bovine whey proteins: opioid and ACE-inhibitory peptides Trends Food Sci Technol 2001 11 347 356

[104] Byun HG Kim SK Purification and characterization of angiotensin I converting enzyme (ACE) inhibitory peptides from Alaska Pollack (*Theragra chalcogramma*) skin Process Biochem 2001 36 1155 1162

[105] Kim SY Je JY Kim SK Purification and characterization of antioxidant peptide from hoki (*Johnius balengerii*) frame protein by gastrointestinal digestion J Nutr Biochem 2007 18 31 38 16563720 10.1016/j.jnutbio.2006.02.006

[106] Mendis E Rajapakse N Kim SK Antioxidant properties of a radical-scavenging peptide purified from enzymatically prepared fish skin gelatin hydrolysate J Agric Food Chem 2005 53 581 587 15686405 10.1021/jf048877v

[107] Jo HY Jung WK Kim SK Purification and characterization of a novel anticoagulant peptide from marine echiuroid worm *Urechis unicinctus* Process Biochem 2008 43 179 184

[108] Rajapakse N Jung WK Mendis E Moon SH Kim SK A novel anticoagulant purified from fish protein hydrolysate inhibits factor XIIa and platelet aggregation Life Sci 2005 76 2607 2619 15769484 10.1016/j.lfs.2004.12.010

[109] Liu Z Dong S Xu J Zeng M Song H Zhao Y Production of cysteine-rich antimicrobial peptide by digestion of oyster (*Crassostrea gigas*) with alcalase and bromelin Food Control 2008 19 231 235

[110] Stensvag K Haug T Sperstad SV Rekdal O Indrevoll B Styrvold OB Arasin 1, a proline-arginine-rich antimicrobial peptide isolated from the spider crab *Hyas araneus* Dev Comp Immuno 2008 32 275 285 10.1016/j.dci.2007.06.002 17658600

[111] Lee TG Maruyama S Isolation of HIV-1 protease-inhibiting peptides from *Thermolysin hydrolysate* of oyster proteins Biochem Bioph Res Co 1998 253 604 608 10.1006/bbrc.1998.9824 9918775

[112] Zampella A Valeria D’Auria M Paloma LG Casapullo A Minale L Debitus C Henin Y Callipeltin A, an anti-HIV cyclic depsipeptide from the new Caledonian lithistida sponge *Callipelta* sp J Am Chem Soc 1996 118 6202 6209

[113] Ford PW Gustafson KR McKee TC Shigematsu N Maurizi LK Pannell LK Williams DE Dilip de Silva E Lassota P Allen TM Soest RV Andersen RJ Boyd MR Papuamides A-D, HIV-inhibitory and cytotoxic depsipeptides from the sponges *Theonella mirabilis* and *Theonella swinhoei* collected in Papua new guinea J Am Chem Soc 1999 121 5899 5909

[114] Rashid MA Gustafson KR Cartner LK Shigematsu N Pannell LK Boyd MR Microspinosamide, a new HIV-inhibitory cyclic depsipeptide from the marine sponge *Sidonops microspinosa* J Nat Prod 2001 64 117 121 11170684 10.1021/np0002379

[115] Oku N Gustafson KR Cartner LK Wilson JA Shigematsu N Hess S Pannell LK Boyd MR McMahon JB Neamphamide A, a new HIV-inhibitory depsipeptide from the Papua new guinea marine sponge *Neamphius huxleyi* J Nat Prod 2004 67 1407 1411 15332865 10.1021/np040003f

[116] Plaza A Gustchina E Baker HL Kelly M Bewley CA Mirabamides A–D, depsipeptides from the sponge *Siliquariaspongia mirabilis* that inhibit HIV-1 fusion J Nat Prod 2007 70 1753 1760 17963357 10.1021/np070306k

[117] Plaza A Bifulco G Keffer JL Lloyd JR Baker HL Bewley CA Celebesides A-C and Theopapuamides B–D, depsipeptides from an Indonesian sponge that inhibit HIV-1 entry J Org Chem 2009 74 504 512 19072692 10.1021/jo802232u PMC2656767

[118] Zampella A Sepe V Luciano P Bellotta F Monti MC D’Auria MV Jepsen T Petek S Adeline MT Laprévôte O Aubertin AM Debitus C Poupat C Ahond A Homophymine A, an anti-HIV cyclodepsipeptide from the sponge *Homophymia* sp J Org Chem 2008 73 5319 5327 18563935 10.1021/jo800583b

[119] Aneiros A Garateix A Bioactive peptides from marine sources: pharmacological properties and isolation procedures J Chromatogr B 2004 803 41 53 10.1016/j.jchromb.2003.11.005 15025997

[120] Andjelic CD Planelles V Barrows LR Characterizing the anti-HIV activity of Papuamide A Mar Drugs 2008 6 528 549 19172193 10.3390/md20080027 PMC2630844

[121] Bhadury P Mohammad BT Wright PC The current status of natural products from marine fungi and their potential as anti-infective agents J Ind Microbiol Biotechnol 2006 33 325 337 16429315 10.1007/s10295-005-0070-3

[122] Yasuhara-Bella J Lu Y Marine compounds and their antiviral activities Antiviral Res 2010 86 231 240 20338196 10.1016/j.antiviral.2010.03.009 PMC7132374

